# Needle in a haystack: Coarse-to-fine alignment network for moment retrieval from large-scale video collections

**DOI:** 10.1371/journal.pone.0320661

**Published:** 2025-05-15

**Authors:** Lingwen Meng, Fangyuan Liu, Mingyong Xin, Siqi Guo, Fu Zou

**Affiliations:** Electric Power Research Institute of Guizhou Power Grid Co. Ltd, Guiyang, China; Prince Mohammad Bin Fahd University, SAUDI ARABIA

## Abstract

Moment retrieval from large-scale video collections aims to search and localize the temporal boundary of a video moment from a collection of numerous videos according to the given natural language query. Existing methods for moment retrieval in a single video is too time-consuming to directly scale to this task due to their sophisticated network architecture. In this paper, we decompose the original problem into two mutually boosting subtasks: video retrieval from video collections and moment retrieval in a single video, and propose the coarse-to-fine alignment network (CFAN) including a video alignment module, a cross-modal interaction module and flow of multi-level coarse-to-fine alignment information. Through the interaction of the multi-level information from two subtasks, our method makes full use of the global contextual information in videos and the fine-grained alignment information between videos and queries. We perform sufficient experiments on three public datasets ActivityNet Captions, Charades-STA and DiDeMo and the evaluation results demonstrate the effectiveness of the proposed CFAN method.

## Introduction

**Fig 1:**
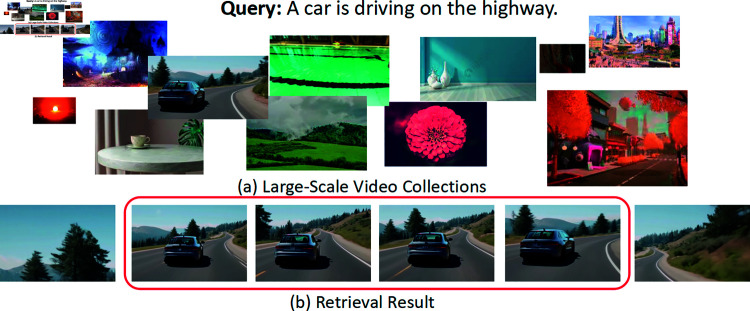
Example of moment retrieval from large-scale video collections.

Video retrieval with natural language [1–6], which aims to search the most relevant video from a large collection of videos, and moment retrieval in video [7–10], where the goal is to localize the temporal boundary of a target moment in a single video, have received significant attention in recent years. Despite the advancements, several limitations persist in prior works. When provided with a textual query, users expect the retrieval system not only to identify videos of interest but also to exclude irrelevant content and pinpoint the most semantically relevant moment accurately. In this paper, we study the task of moment retrieval from large-scale video collections, as a natural and essential extension of prior tasks, aiming to identify a video moment from a large collection of videos according to the given natural language query, as the example in Fig 1 shows.

Localizing the temporal boundary of a moment from video collections considering both efficiency and accuracy is much more challenging than prior tasks. A simple way is to scale those methods of moment retrieval in a single video [3–5, 11–13] to the large video collections and generate confidence score for the predicted moment in each video. However, due to the sophisticated network architecture of those proposed methods, generating candidate moments and its corresponding confidence score video by video are expensive, time-consuming and unrealistic. A smarter way is to efficiently search a most relevant video for moment retrieval but it is also greatly limited by video-level alignment and ignores the benefits of fine-grained alignment. Another way is to break a video into a short sequence of clips and align the clips of target moment to the given textual query with a clip-alignment loss [14]. Despite faster retrieval, coarsely aligning the clips of target moment with query ignores the global contextual information of the video, leading to insufficient understanding of video contents. Also, treating different clips in the same video separately raises two crucial challenges: semantic misalignment and structural misalignment [12], which is not helpful for accurate retrieval.

To tackle these challenges and achieve an optimal balance between speed and accuracy, we propose the Coarse-to-Fine Alignment Network (CFAN). This approach decomposes the original problem into two interrelated and mutually enhancing subtasks: video retrieval from a video collection and moment retrieval within a single video. Instead of merely selecting a video for moment prediction or directly searching for the target clip in a clip database [14], we first efficiently retrieve a small candidate set of videos by learning a shared visual-semantic space for video alignment. We then design an advanced cross-modal interaction mechanism to refine the fine-grained alignment between candidate proposals, video frames, and the query. The video alignment step also serves as auxiliary guidance to enhance this process. The fine-grained alignment further acts as a guided gating mechanism, emphasizing key content relevant to the query and refining the learned visual-semantic space. By integrating multi-level coarse-to-fine alignment information across the two subtasks, our method fully leverages both the global contextual information of videos and the detailed correspondence between frames and the query. This comprehensive interaction enables more accurate and efficient retrieval.

Specifically, to learn the common visual-semantic space, we devise a video alignment module where the multi-head self attention mechanism [15] is plugged into the trainable generalized Vector of Locally Aggregated Descriptors (VLAD) layer [16] to learn the spatio-temporal descriptors, and the sum of residuals from different cluster center is aggregated by mean pooling to obtain the visual-semantic embeddings for query and video. To explore the fine-grained alignment among the candidate proposals, frames and the query, we propose a cross-modal interaction module including attention aggregation, cross gate guided by video alignment and BiGRU [17] to obtain the cross-modal representations for frame and proposal alignment. Moreover, the frame alignment information and the hardest negative samples for video alignment are applied to fine tune the video alignment module and improve the learned visual-semantic space for correlation re-estimation. In total, our contributions can be summarized as follows:

- We decompose the original retrieval task into two mutually boosting subtasks, which considers both the global contextual information of videos and the fine-grained information between videos and queries.
- We propose a novel and effective coarse-to-fine alignment network including a video alignment module, a cross-modal interaction module, and the interaction of multi-level coarse-to-fine alignment information for moment retrieval from large-scale video collections, which can be trained in an end-to-end manner.
- We perform sufficient experiments on three public datasets: ActivityNet Captions [18], Charades-STA [7] and DiDeMo [8], and validate the effectiveness of our CFAN method.

## Related work

### Video retrieval with natural language

With a natural language query, video retrieval aims to retrieve a specific video from a candidate set of videos. Most methods [3–5, 19–23] maximize the similarity score between video and its corresponding caption while minimize the score between negative pairs by encoding both video and text into a common visual-semantic space. For fine-grained video and query encoding, Xu *et al*. [3] propose a compositional semantics language model with the dependency-tree structure and a deep video model to extract visual features. Otani *et al*. [4] leverage the web image search results to disambiguate fine-grained visual concepts in the query sentence and compute the sentence embeddings. Yu *et al*. [20] propose a trainable high-level concept word detector as useful semantic priors and develop an attention mechanism that selectively focuses on the detected concept words and fuse them with word encoding. Mithun *et al*. [5] and Miech *et al*. [22] both utilize multi-modal features (e.g. motion, audio) from a video for more robust video understanding. Shen *et al*. [24] used contrastive learning and Transformer model to effectively exploit the long dependency between video and text in cross-modal video-text retrieval task, thereby improving the accuracy and efficiency of retrieval. Zhang *et al*. [25] proposed an asymmetric co-attention network for video clip and text alignment, which effectively handles the information asymmetry between video and text through a specially designed contrastive loss function, achieving excellent performance on multiple benchmark datasets. The Hierarchical Sequence Embedding (HSE) [21] exploit both low-level and high-level correspondences in the hierarchically semantic spaces, and the Dual Encoding Network [23] proposes multi-level encodings including global, local and temporal patterns in both videos and sentences to learning better shared representations. In this paper, we extend the video retrieval to the moment retrieval from large-scale video collections and leverage the fine-grained alignment to improve the performance of video retrieval.

### Moment retrieval in video

According to the given textual query, moment retrieval aims to identify the temporal boundary of the most semantically-matching moment in the video. Ealry methods [7–9] sample the moment candidates with multi-scale slide windows, map their visual features and the textual features of the query into a joint semantic space and maximize the similarity score of each positive moment-query pair. For better understanding and modeling of both query and video, Liu *et al*. [26] propose a language-temporal attention network that encodes the temporal context information to comprehend query descriptions. Xu *et al*. [11] employ an early fusion approach to generate clip proposals and further consider video captioning as an auxiliary task to learn better representations. Zhang *et al*. [12] devise an iterative graph adjustment network to exploit the graph-structured moment relations in the videos. Chen *et al*. [27] proposes a cross-modal semantic alignment and contrastive learning approach to improve the accuracy and efficiency of video moment retrieval. By enhancing the semantic alignment between video and text, the model achieves more accurate moment localization. Gao *et al*. [28] introduces a time localization method based on attention mechanism, which realizes accurate retrieval of video moments through fine-grained temporal modeling. Zhao *et al*. [29] uses a graph network to build the context representation in the video, which improves the effect of moment retrieval through the combination of semantic and structural information. Zhang *et al*. [13] propose a multi-head self-attention mechanism to capture the long-range dependencies in videos and a graph network to exploit the syntactic dependencies in the queries. Some methods [30–32] also study this task in a weakly-supervised setting, which requires only video-level annotations for training.

As natural extensions of moment retrieval in video, Zhang *et al*. [33] propose the self-attention interaction localizer (SAIL) to localize unseen activities in video via an image query. Yuan *et al*. [34] propose a novel graph convolved video thumbnail pointer (GTP) to dynamically select and concatenate multiple video clips from an original video via a textual query. Escorcia *et al*. [14] devise a Clip Alignment with Language (CAL) model to localize the relevant moment in a large collection of videos rather than one single video. In this paper, we also study the task of moment retrieval in video collections, which is more practical and useful than moment retrieval in a single video.

## Proposed method

### Problem statement and decomposition

Given a natural language query $q=\{q_{i}{\}}_{i=1}^{n_{q}}$, our goal is to search a video $v=\{v_{i}{\}}_{i=1}^{n_{v}}$ from a video collection V, and further localize the temporal boundary $\hat{\tau }=\left(\hat{s},\hat{e}\right)$ of the moment that is the most relevant to the query, where $q_{i}$ is the word embedding of the *i*-th word in the query, *n*_*q*_ is the total number of words of the query, **v** is a video in the collection V, $v_{i}$ is the pre-extracted feature of the *i*-th frame of **v**, and $n_{v}$ is the total number of frames of the video.

**Fig 2:**
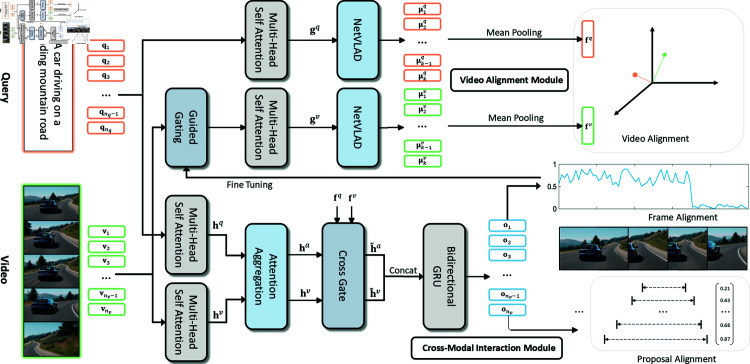
The overview framework of the coarse-to-fine alignment network. The CFAN consists of a video alignment module to learn the common visual-semantic space, a cross-modal interaction module to explore the fine-grained alignment among frames, proposals and video. Also, the multi-level coarse-to-fine alignment information flows between modules to make full use of both the global contextual information and fine-grained alignment information.

To address the issues of prior works mentioned above, exhibit a good speed-accuracy trade-off and make use of global and fine-grained information, we decompose the original problem into two mutually boosting subtasks: video retrieval from video collections and moment retrieval in a single video. Following our idea, we devise an effective and novel coarse-to-fine alignment network, as shown in Fig 2. Specifically, we first develop a video alignment module to retrieve a small candidate set of videos efficiently by learning a common visual-semantic space. We then develop a sophisticated cross-modal interaction to explore the fine-grained alignment among the candidate proposals, frames and the query, and the visual-semantic embeddings learned before can be employed as extra guidance information. Moreover, we leverage the fine-grained frame alignment and the hardest negative samples for video alignment to improve the common visual-semantic space and boost the video alignment module.

### Video alignment

To learn a common visual-semantic space for video alignment, we devise a dual video alignment module where the multi-head self attention mechanism [15] is plugged into the trainable generalized Vector of Locally Aggregated Descriptors (VLAD) layer, also named NetVLAD, [16] to learn the spatio-temporal descriptors, and the sum of residuals from different centers is aggregated by mean pooling to obtain the visual-semantic embeddings for query and video.

**Multi-head self attention.** The self-attention mechanism is able to learn the global interaction between each pair of items in a sequence while the multi-head setting ensure the sufficient understanding of complex information. Specifically, we first employ the multi-head self attention mechanism to absorb the global contextual information for query and video modeling. The contextual representations $g^{v}=\{g_{i}^{v}{\}}_{i=1}^{n_{v}}$ of video **v** can be represented as follows:

$$g^{v}=\text{MultiHead}(v)+v,$$ (1)

**NetVLAD.** Given the encoded sequence $g^{v}$ and the trainable cluster centers $c=\{c_{j}{\}}_{j=1}^{k}$ where $g_{i}^{v},c_{j}\in {ℛ}^{d}$, and *k* is the number of cluster center, to obtain the spatio-temporal descriptors, the trainable VLAD accumulates the residuals between local descriptors and multiple cluster centers by a differentiable and soft assignment, denoted by

$$\mu_{j}^{v}=\sum \sum_{i=1}^{n_{v}}a_{ij}(g_{i}^{v}-c_{j}),\;a_{i}=\text{softmax}(W^{k}g_{i}^{v}+b^{k}),$$ (2)

where $W^{k}\in {ℛ}^{k\times d}$, $b^{k}\in {ℛ}^{k}$, $a_{i}$ is the soft assignment vectors of descriptor $h_{i}^{v}$ for *k* cluster centers and $a_{ij}$ the assignment of descriptor $g_{i}^{v}$ to the *j*-th cluster center.

The visual-semantic embedding of video $f^{v}\in {ℛ}^{d}$ can be aggregated by mean pooling of spatio-temporal descriptors $\mu^{v}$. Similarly, we can obtain the visual-semantic embedding of query **f**^*q*^ ∈ *ℛ*^*d*^ by dual operation. With **f**^*q*^ and $f^{v}$, we can build a candidate set of videos $V_{c}$ by selecting top-K relevant videos based on cosine similarity cos(·) of two embeddings.

### Cross-modal interaction

To explore the fine-grained alignment among the proposals, frames and the query, we propose a cross-modal interaction module including attention aggregation, cross gate guided by visual-semantic embeddings and BiGRU [17] to obtain the cross-modal representations for further alignment. Similarly, we can obtain the global contextual representations for video and query by multi-head self attention, denoted as $h^{v}=\{h_{i}^{v}{\}}_{i=1}^{n_{v}}$ and $h^{q}=\{h_{i}^{q}{\}}_{i=1}^{n_{q}}$.

**Attention aggregation.** We employ a usual attention mechanism to aggregate the contextual representations of query for each frame, denoted by

$$e_{ij}=w^{T}\text{tanh}(W_{1}^{a}h_{i}^{v}+W_{2}^{a}h_{j}^{q}+b^{a}),\;h_{i}^{a}=\sum \sum_{j=1}^{n_{q}}\frac{\text{exp}(e_{ij})}{\sum_{j=1}^{n_{q}}\text{exp}(e_{ij})}h_{j}^{q},$$ (3)

where $W_{1}^{a},W_{2}^{a}\in {ℛ}^{d\times d}$, $w,b^{a}\in {ℛ}^{d}$, $e_{ij}$ is the attention score between $h_{i}^{v}$ and $h_{j}^{q}$, and $h^{a}=\{h_{i}^{a}{\}}_{i=1}^{n_{v}}$ is the aggregated result.

**Cross Gate.** As an extension of ordinary cross gate [35], we introduce the visual-semantic embeddings of query and video, $f^{q}$ and $f^{v}$ as high-level information for guidance and further fusion, denoted by

$${\tilde{h}}_{i}^{a}=(h_{i}^{a}+f^{q})⊙\sigma (W_{1}^{c}h_{i}^{v}+W_{2}^{c}f^{v}),\;{\tilde{h}}_{i}^{v}=(h_{i}^{v}+f^{v})⊙\sigma (W_{3}^{c}h_{i}^{a}+W_{4}^{c}f^{q}),$$ (4)

where $W_{1}^{c},W_{2}^{c},W_{3}^{c},W_{4}^{c}\in {ℛ}^{d\times d}$, and ${\tilde{h}}^{a}$ and ${\tilde{h}}^{v}$ are gated representations of query and video separately. Taking the concatenation of ${\tilde{h}}^{a}$ and ${\tilde{h}}^{v}$ as the input of BiGRU, we can obtain the final cross-modal representations $o=\{o_{i}{\}}_{i=1}^{n_{v}}$.

**Proposal alignment.** Based on the cross-modal representations **o**, we sample a fixed number of candidate proposals according to a set of ratios $\{r_{i}{\}}_{i=1}^{n_{r}}$ at each time step and score all of them in one single pass, where $r_{i}\in [0,1]$ and *n*_*r*_ is the number of candidate proposals at each time step. Through dense sampling, the set of candidate proposals can be denoted as $\{\{(i-r_{j}*n_{v},i+r_{j}*n_{v}){\}}_{j=1}^{n_{r}}{\}}_{i=1}^{n_{v}}$, where $i-r_{j}*n_{v},i+r_{j}*n_{v}$ are the temporal boundaries of the *j*-th proposal at *i*-th time step. The scoring for proposal alignment can be denoted as follows:

$$s_{i}^{m}=\sigma (W_{2}^{m}(\text{tanh}(W_{1}^{m}o_{i}+b_{1}^{m}))+b_{2}^{m}),\;$$ (5)

where $W_{1}^{m}\in {ℛ}^{d\times d}$, $W_{2}^{m}\in {ℛ}^{n_{r}\times d}$, $b_{1}^{m}\in {ℛ}^{d}$, $b_{2}^{m}\in {ℛ}^{n_{r}}$ and $s_{i}^{m}\in {ℛ}^{n_{r}}$ is the alignment score at *i*-th time step.

**Frame alignment.** In a similar way, for frame alignment, we compute a score of query-relevance for each frame according to the cross-modal representations **o**, denoted by

$$h_{i}^{f}=\text{tanh}(W_{1}^{f}o_{i}+b_{1}^{f}),\;s_{i}^{f}=\sigma (W_{2}^{f}h_{i}^{f}+b_{2}^{f}),\;$$ (6)

where $W_{1}^{f}\in {ℛ}^{d\times d}$, $W_{2}^{f}\in {ℛ}^{1\times d}$, $b_{1}^{f}\in {ℛ}^{d}$, $b_{2}^{f}\in ℛ$ and $s_{i}^{f}\in ℛ$ is the alignment score for *i*-th frame of the video.

**Guided gating.** The fine-grained frame alignment can not only be regarded as an auxiliary task to enhance proposal alignment, but also be leveraged as the guided gating information of the predicted moment to highlight the key content relevant to the query and weaken the background content in video. The query-aware video $\tilde{v}$ can be computed as follows:

$${\tilde{\text{v}}}_{i}=v_{i}⊙\sigma (W^{g}h_{i}^{f}+b^{g}),\;$$ (7)

where $W^{g}\in {ℛ}^{d\times d}$, $b^{g}\in {ℛ}^{d}$ and ${\tilde{\text{v}}}_{i}$ is the visual feature of *i*-th query-aware frame.

Then the query-aware video $\tilde{\text{v}}$ is employed as training data to fine tune the video alignment module and improve the learned visual-semantic space. We compute the similarity scores of video and query based on both the original and improved common space, given by

$$\text{s}im(v,q)=\text{cos}(f^{v},f^{q})+\text{cos}({\tilde{\text{f}}}^{v},{\tilde{\text{f}}}^{q}),$$ (8)

where ${\tilde{\text{f}}}^{v}$ and ${\tilde{\text{f}}}^{q}$ are the improved visual-semantic embeddings of videos and queries given by the fine-tuned video alignment module.

### Training

In this section, we describe the training strategy and the loss function we devised. To provide a more intuitive explanation of our method, we present the training pseudocode of the proposed CFAN in Algorithm 1.

**Algorithm 1. Training process of the proposed CFAN**



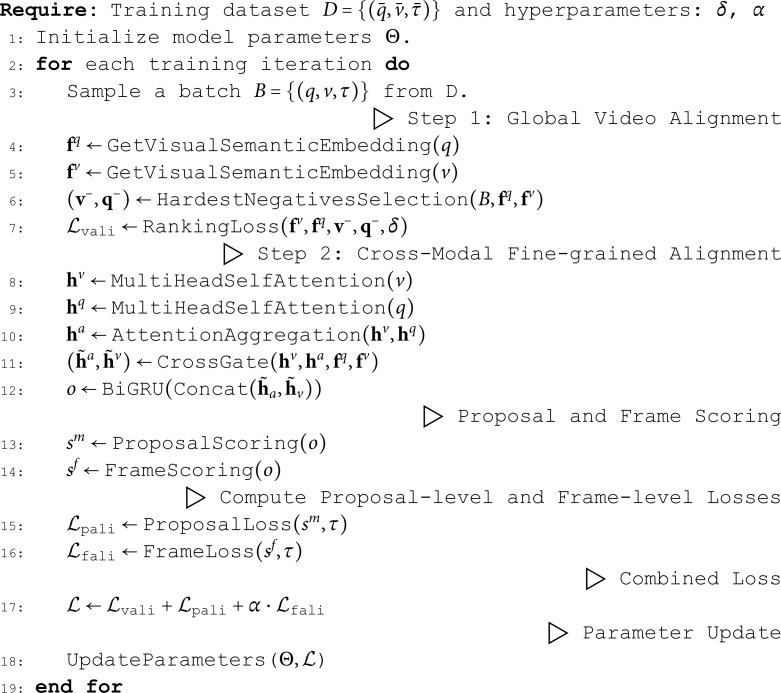



**Video alignment loss.** We first adopt a video alignment loss to minimize the distance between positive pairs of video and query while maximize the distance of negative pairs based on the bidirectional max-margin ranking loss [36], denoted by

$${ℒ}_{vali}=\text{max}(0,\delta +{\text{V}}_{\theta }(v^{-},q)-{\text{V}}_{\theta }(v,q))\;+\text{max}(0,\delta +{\text{V}}_{\theta }(v,q^{-})-{\text{V}}_{\theta }(v,q)),$$ (9)

where $v^{-}$ and $q^{-}$ represents a negative video for **q** and a negative query for **v** respectively, ${\text{V}}_{\theta }(\cdot )$ is the cosine distance of the visual-semantic embeddings of the given query and video and δ is the margin.

**Proposal alignment loss.** During training, we determine the label of a candidate proposal according to its temporal IoU with the target moment. Moreover, to keep the number of positive and negative proposals in a fixed ratio, we mark some positive proposals with lower IoU as negative and assign zero to their labels. Given a video **v**, the proposal alignment loss ${ℒ}_{pali}$ is defined as:

$${ℒ}_{pali}=-\frac{1}{n_{v}n_{r}}\sum \sum_{i=1}^{n_{v}}\sum \sum_{j=1}^{n_{r}}(I_{ij}\text{log}(s_{ij}^{m})\;+(1-{\text{I}}_{ij})\text{log}(1-s_{ij}^{m})),$$ (10)

where $s_{ij}^{m}$, ${\text{I}}_{ij}$ are separately the confidence score and the discretized label for the *j*-th proposal at *i*-th time step,

**Frame alignment loss.** For frame alignment, we hope the higher scores are assigned to frames in the target moment while the lower scores to those frames outside the target moment. Given a video **v**, the frame alignment loss ${ℒ}_{fali}$ is computed as follows:

$${ℒ}_{fali}=-(\frac{1}{e-s+1}\sum \sum_{i\in [s,e]}\text{log}(s_{i}^{f})\;+\frac{1}{n_{v}-e+s-1}\sum \sum_{i\notin [s,e]}\text{log}(1-s_{i}^{f})),$$ (11)

We eventually employ a multi-task loss considering multi-level coarse-to-fine alignment to train our CFAN model, denoted by

$$ℒ={ℒ}_{vali}+{ℒ}_{pali}+\alpha {ℒ}_{fali},$$ (12)

where α is the trade-off hyper-parameter.

**Hardest negative samples.** Besides leveraging the fine-grained alignment information by the guided gating mechanism, we also introduce the hardest negative examples for video alignment to strengthen the training data [37] when fine tuning the video alignment module for correlation re-estimation. The loss function is a modified version of ${ℒ}_{vali}$ that selects $v^{-}$ and $q^{-}$ from the hardest negative samples instead of randomly selecting a negative sample in the minibatch.

During evaluation, we can build a candidate set $V_{c}$ efficiently based on the cosine similarity $\text{cos}(f^{v},f^{q})$ where the visual-semantic embeddings $f^{v}$ over all videos can be pre-computed, and select a video according to the more accurate similarity score sim(v,q) that considering both the global contextual information and fine-grained alignment information for further moment retrieval. Moreover, since the fine-grained but time-consuming re-estimation and moment retrieval are limited to the small candidate set $V_{c}$, our method can effectively achieve a balance between speed and accuracy.

## Experiments

In this section, we first introduce the datasets we used, the implementation details of our method and the evaluation criteria, and then compare our method with some existing state-of-the-art methods. Next, we conduct several ablation experiments to explore the impact of different steps of our algorithm. We also provide some quantitative results of retrieval to prove the effectiveness of our method.

### Datasets

Our experiments are conducted on the ActivityNet Captions, DiDeMo, and Charades-STA datasets. They are publicly available datasets widely used for video understanding and video-text retrieval tasks.

**ActivityNet Captions** [18]. The ActivityNet Captions dataset connects over 20,000 untrimmed videos from the ActivityNet [38] dataset to temporally annotated sentences. Each sentence describes an event occurring within a unique segment of the video. On average, each video contains 3.65 temporally localized sentences, leading to a total of approximately 100,000 sentences. The length of each sentence averages 13.48 words and covers 36 seconds or about 31% of the video’s total duration. These sentences, when considered together, account for 94.6% of the entire video length. Furthermore, 10% of the temporal descriptions overlap, reflecting the co-occurrence of events. The dataset emphasizes action-centric descriptions, with a higher prevalence of verbs and pronouns compared to other datasets such as Visual Genome [18].

**DiDeMo** [8]. The DiDeMo dataset comprises over 10,000 25-30 second personal videos sourced from the YFCC100M dataset [39]. It includes 41,206 moment-query pairs split into training (33,005), validation (4,180), and test (4,021) subsets. Each video is segmented into 5-second intervals, with moments consisting of one or more consecutive segments. The dataset is specifically designed to localize moments with natural language descriptions. The descriptions in DiDeMo are verified to ensure they refer to specific moments in the video, making it one of the largest and most diverse video-language datasets for temporal localization. The dataset contains 26,892 moments, with descriptions provided by multiple annotators. The videos focus on personal activities, with detailed annotations including camera movements and time transitions.

**Charades-STA** [7]. The Charades-STA dataset builds upon the Charades dataset [40] and includes 12,408 moment-query pairs for training and 3,720 for testing. Charades originally provides video-level descriptions, but Charades-STA adds clip-level temporal annotations. A semi-automatic method was developed to generate these annotations: long sentences were split into sub-sentences, and temporal annotations were assigned to these sub-sentences by matching keywords with activity categories. Each sub-sentence is associated with a specific time span. In total, Charades-STA contains 13,898 clip-sentence pairs for training, 4,233 for testing, and 1,378 complex sentence queries for testing. The dataset focuses on household activities, and most descriptions follow a syntactic pattern, with sub-sentences connected by conjunctions like “then,” “while,” and “and.”

The primary quantitative information regarding the aforementioned three datasets is presented in Table 1.

**Table 1:** The details of the ActivityNet Captions, DiDeMo and Charades-STA Dataset.eak Table includes number of videos, number of queries, average video length and average query length.

Dataset	ActivityNet Captions [18]	DiDeMo [8]	Charades-STA [7]
Num. Video	20,000	10,642	6,067
Num. Query	100,000	41,206	13,760
Avg. Video Len	120 secs	29 secs	31 secs
Avg. Query Len	13.48 words	7.98 words	7.26 words

### Implementation details

We train the CFAN model in an end-to-end manner using the Pytorch framework with four NVIDIA 3090 GPUs. Specifically, we apply the pre-trained Glove word2vec [41] to extract initial textual features for each query. For ActivityNet Captions and Charades-STA, we employ pre-trained 3D-ConvNet [42] to extract initial visual features and use PCA to reduce the data dimension for each video. For DiDeMo, we follow the prior work [8] that uses the pre-trained VGG [43] to extract RGB features and a competitive activity recognition model [44] to extract optical flow features. The RGB features and optical flow features are fused by concatenation. For model setting, we set the hidden size of model *d* to 512. The number of clusters for query and video in NetVLAD is set to 16. To sample the candidate moments at each time step, the sample ratio is set to {0.04,0.08,0.16,0.24,0.32,0.49} for ActivityNet Captions, to {0.04,0.08,0.12,0.16,0.24} for Charades-STA and to {0.0,0.08,0.16,0.25,0.33} for DiDeMo, and the illegal moments are removed from the candidate set. The trade-off hyper-parameter α is set to 0.5. The whole CFAN model is trained by Adam optimizer with learning rate 0.0006 for training and 0.0004 for fine tuning. The size of candiate set $\left|V_{c}\right|$ is set to 5.

### Evaluation criteria

Following [7], we adopt the “R@n, IoU=m” accuracy as the evaluation metric of moment retrieval in video collections, where average recall over all test queries is computed by determining whether one of the top-n predicted moments has Intersection over Union (IoU) larger than m. Moreover, we also compute the “Recall@K” as the criteria for sentence-to-video retrieval, where average recall over all test queries is determined by whether one of the top-K returned videos is the target video. “R@n, IoU=m” can be regarded as a criterion for moment-level retrieval evaluation, while “Recall@K” as a criterion for video-level retrieval evaluation. Note that the DiDeMo dataset contains multiple temporal annotations from different annotators for each description. Following [14], the predicted moment must have IoU larger than the specified m with at least two ground truth moments.

### Performance comparison

We compare our CFAN method with some existing methods to verify the effectiveness.

**Chance.** [14] For chance, moments across all videos are sampled and returned based on a uniform distribution.

**Moment Prior.** [14] The moment prior method samples a video from a uniform distribution and return a moment based on the moment frequency prior [8].

**MCN.** [8] The MCN method for single video moment retrieval is scaled to the large-scale video collections by enumerating all the candidate moments exhaustively and returning the moment with the highest score.

**CAL.** [14] To retrieve a specific moment from video collections, the CAL method splits video into clips, builds a clip database and minimize the squared-Euclidean distance between moment’s visual features and language feature.

**M-DETR.** [45] MDETR is an end-to-end modulated detector that conditions object detection on raw text queries, integrating text and image modalities early in its transformer-based architecture. Pre-trained on 1.3M text-image pairs, it achieves remarkable performance on tasks like phrase grounding and referring expression comprehension while effectively addressing the long-tail problem in object categories through few-shot fine-tuning.

**UniVTG.** [46] UniVTG is a unified framework that consolidates diverse video temporal grounding tasks and label types into a single model, enabling large-scale pretraining, zero-shot generalization, and flexible adaptation across various VTG tasks such as moment retrieval, highlight detection, and video summarization.

**QD-DETR.** [47] QD-DETR is a query-dependent detection transformer designed for video moment retrieval and highlight detection, enhancing query-video relevance by explicitly injecting query context via cross-attention and training on negative query-video pairs to improve saliency estimation. It features an input-adaptive saliency predictor and achieves satisfying results.

The overall performance evaluation results of our CFAN method and other baselines on the ActivityNet Captions, DiDeMo and Charades-STA datasets are shown in Table 2.

**Table 2:** Performance evaluation results on the ActivityNet Captions, DiDeMo and Charades-STA datasets. We show the results with the mertic “R@n, IoU=m” where n ∈ {1, 10, 10}, m ∈ {0.5, 0.7}.

	Method	ActivityNet Captions [18]			DiDeMo [8]			Charades-STA [7]		
		R@1	R@10	R@100	R@1	R@10	R@100	R@1	R@10	R@100
IoU=0.5	Chance	0.00	0.02	0.18	0.00	0.10	1.99	0.01	0.09	1.09
	Moment Prior	0.01	0.05	0.47	0.02	0.22	2.34	0.02	0.17	1.63
	MCN	0.12	0.75	4.54	0.88	5.16	26.23	0.13	0.96	6.05
	CAL	0.21	1.32	6.82	0.97	6.15	28.06	0.23	1.39	7.03
	M-DETR	0.35	1.97	8.26	1.32	6.79	26.97	0.15	0.34	2.98
	UniVTG	0.64	2.48	10.64	1.68	8.65	27.54	0.25	0.39	2.85
	QD-DETR	0.94	4.49	13.87	1.88	9.54	28.24	0.38	0.78	6.84
	**CFAN**	**1.85**	**10.43**	**27.64**	**2.36**	**10.52**	**29.75**	**0.56**	**1.72**	**9.33**
IoU=0.7	Chance	0.00	0.01	0.06	0.00	0.02	0.64	0.00	0.03	0.39
	Moment Prior	0.00	0.03	0.26	0.02	0.17	1.99	0.01	0.05	0.56
	MCN	0.07	0.48	3.04	0.58	4.12	21.03	0.08	0.63	4.24
	CAL	0.12	0.89	4.79	0.66	4.69	22.89	0.12	1.00	4.91
	M-DETR	0.27	1.59	8.97	1.24	4.87	21.77	0.07	0.24	2.14
	UniVTG	0.59	2.17	10.11	1.39	7.04	21.68	0.09	0.31	2.68
	QD-DETR	0.74	3.98	12.86	1.52	7.44	23.54	0.11	0.71	3.98
	**CFAN**	**1.12**	**6.15**	**15.88**	**1.54**	**7.59**	**23.94**	**0.38**	**1.31**	**5.81**

Compared with all the other baselines, our method achieves huge improvement across all datasets, which demonstrates the effectiveness of our method including the interaction of multi-level coarse-to-fie alignment and the CFAN framework for moment retrieval from video collections. In particular, the results of “R@1, IoU=0.5” and “R@1, IoU=0.7” increase by 100%–780% over the prior CAL method for our task. Note that due to a large number of candiate moments, the evaluation results are low for all the baselines based on clip retrieval, especially Chance, indicating the difficulty of our task and further illustrating the effectiveness of our method.

As results shown in Table 2, our method also significantly outperforms the CAL method by a large margin on ActivityNet Captions dataset, especially on the criteria “R@100, IoU=0.5” and “R@100, IoU=0.7”, which indicates the superiority of our method on the ActivityNet Captions dataset. As shown in Table 1, there are more videos on the ActivityNet Captions dataset, and each video is much longer over the other two datasets, leading to a large number of candiate clips for the CAL method. Searching a specific clip relevant to the query in such a big clip database is extremely difficult and is susceptible to noise of clips from other videos. Instead, our method first retrieve a candiate set of videos based on the high-level visual-semantic space, and further leverage the fine-grained alignment information of moment retrieval for correlation re-estimation, which greatly reduces the noise of clips from other videos, and further speed up the retrieval.

Moreover, we can find that the overall evaluation results on the Charades-STA are lower than the other two datasets, and meanwhile our method only obtains a smaller absolute improvement over CAL for “R@1, IoU=0.5” and “R@n, IoU=0.7”. That is because there are more similar videos that describe the same activity of humans in a similar sentence pattern, leading to more noise for retrieval of the target video. Also, longer sentence can provide more information while the average length of descriptions on the Charades-STA dataset is shorter than the other two datasets, as shown in Table 1.

### Ablation study

To verify the effectiveness of and each module in our CFAN model and the interaction of multi-level coarse-to-fine alignment information, we next conduct some ablation experiments. Specifically, we modify our method to generate ablation models as follows:

**CFAN(w/o. RE).** We simply compute the similarity score sim(v,q) by $\text{cos}(f^{v},f^{q})$ without using the improved visual-semantic embeddings for correlation re-estimation.

**CFAN(w/o. HN).** Instead of selecting negative videos and queries from the hardest samples, we still randomly choose a negative sample to fine tune the video alignment module for correlation re-estimation.

**CFAN(w/o. FA).** We remove the frame alignment loss ${ℒ}_{fali}$ and the frame alignment information is unused for proposal alignment enhancement and guided video gating.

**CFAN(w/o. CG).** Instead of integrating the high-level visual embeddings into the cross gate, we apply the ordinary cross gate taking $h^{a}$ and $h^{v}$ as input without extra guidance.

The evaluation results of ablation models on ActivityNet Captions and DiDeMo datasets are shown in Tables 3 and 4 respectively. It can be observed that all the ablation models still achieve better performance than the baselines, demonstrating the effectiveness of the whole framework again. Compared with other ablation models, the CFAN(w/o. RE) achieves the worst performance on both the “Recall@1” and “Recall@10” criterion, indicating that the improved common visual-semantic space are helpful for the re-estimation of correlation between video and query. The full model outperforms the CFAN(w/o. HN), which demonstrates that incorporating the hardest negative samples can effectively boost the video-level alignment. The results of CFAN(w/o. FA) without the frame alignment loss also show a decrease in performance, demonstrating that the frame alignment information can effectively boost the proposal alignment and the video alignment for more accurate retrieval. Moreover, the CFAN(w/o. CG) achieves worse performance than the full model, indicating the high-level visual embeddings can considered as the guidance for fine-grained alignment. The evaluation results of CFAN(w/o. FA) and CFAN(w/o. CG) verify the effectiveness of the interaction of multi-level coarse-to-fine alignment information.

**Table 3:** Evaluation results of ablation study on the ActivityNet Captions dataset. n ∈ {1, 10}, m ∈ {0.5, 0.7} and K ∈ {1, 10}

Method	R@1	R@1	Recall	R@10	R@10	Recall
	IoU=0.5	IoU=0.7	@1	IoU=0.5	IoU=0.7	@10
w/o. RE	1.73	0.94	3.78	9.84	5.96	21.23
w/o. HN	1.75	0.99	3.88	10.22	6.06	21.87
w/o. FA	1.76	1.02	4.09	10.27	6.08	22.11
w/o. CG	1.76	1.03	4.15	10.31	6.03	22.28
**full**	**1.85**	**1.12**	**4.27**	**10.43**	**6.15**	**22.46**

**Table 4:** Evaluation results of ablation study on the DiDeMo dataset. n ∈ {1, 10}, m ∈ {0.5, 0.7} and K ∈ {1, 10}

Method	R@1	R@1	Recall	R@10	R@10	Recall
	IoU=0.5	IoU=0.7	@1	IoU=0.5	IoU=0.7	@10
w/o. RE	1.89	1.29	4.75	9.65	6.91	23.65
w/o. HN	2.04	1.31	5.12	9.77	7.09	24.02
w/o. FA	2.07	1.36	5.22	10.20	7.31	24.92
w/o. CG	2.16	1.32	5.27	9.72	7.16	24.77
**full**	**2.36**	**1.54**	**5.39**	**10.52**	**7.59**	**25.09**

## Limitations and future work

The proposed method has two main limitations. First, it relies heavily on high-quality labeled training data, which is costly and labor-intensive to acquire. Second, its task-specific feature extraction and alignment strategies are sensitive to distributional shifts in video content and language expressions, limiting generalizability across domains. Future work could explore self-supervised or weakly supervised learning to reduce dependence on annotations and enhance domain adaptation techniques for greater robustness. Additionally, improving query comprehension and fine-grained alignment mechanisms could help address challenges in handling complex queries and capturing subtle video details.

## Conclusion

In this paper, we study the task of moment retrieval from large-scale video collections which aims to search and localize the temporal boundary of a moment from a collection of numerous videos according to the given textual query. To make full use of both the global contextual information of videos and the fine-grained alignment information between videos and queries, we decompose the original problem into two mutually boosting subtasks: video retrieval from video collections and moment retrieval in a single video, and propose the coarse-to-fine alignment network (CFAN) that leverages multi-level coarse-to-fine alignment information. The sufficient experiments are performed on three datasets ActivityNet Captions, DiDeMo and Charades-STA and the evaluation results demonstrate the effectiveness of the proposed method.
